# Successful treatment with laparoscopy-assisted surgery for ileal perforation due to an ingested foreign body: A report of two cases

**DOI:** 10.1016/j.ijscr.2019.10.018

**Published:** 2019-10-17

**Authors:** Soichi Ito, Yuma Tsuchitani, Souhei Hashimoto, Takuji Uemura, Kazunori Katsura, Takayuki Abe, Koichiro Sato, Hirotaka Kato

**Affiliations:** Department of Surgery, Iwate prefectural Iwai hospital, 17 Odaira, Kozenji, Ichinoseki, Iwate, 029-0192, Japan

**Keywords:** CT, computed tomography, FB, foreign body, Small bowel perforation, Foreign body, Laparoscopy-assisted surgery, Case report

## Abstract

**Introduction:**

Presently, the advantage of laparoscopic surgery is widely recognized and it is gaining popularity not only in elective abdominal surgeries but also in various kinds of emergent abdominal surgeries. This report describes two patients diagnosed with ileal perforation due to an ingested foreign body (FB)—a rare abdominal emergency—who were treated laparoscopically.

**Presentation of case:**

Both patients were brought in by an ambulance to our hospital, with complaints of increasing lower abdominal pain. Computed tomography scan revealed a small bowel perforation due to an ingested FB in both patients, and laparoscopy-assisted partial ileal resection was immediately performed. Their postoperative recoveries were uneventful.

**Discussion:**

Both patients with this rare abdominal emergency were successfully treated with laparoscopic surgery. Its feasible diagnostic and therapeutic abilities and decreased invasiveness contributed to their uneventful and fast recoveries. Extracorporeal handling of the affected small bowel via mini-laparotomy was useful in this setting as it was safe, fast, and cost-effective.

**Conclusion:**

Laparoscopic surgery is effective, even for this rare abdominal emergency. Moreover, laparoscopy-assisted surgery accompanying mini-laparotomy is a rational treatment approach, especially for this condition.

## Highlights

• Small bowel perforation by an ingested foreign body (FB) is a rare abdominal emergency.
• Computed tomography scan is most effective but imperfect for diagnosis.
• Inquiring about FB ingestion and a high index of suspicion are very important.
• Laparoscopic surgery is effective even in this rare abdominal emergency.
• Extracorporeal handling of the affected small bowel is useful in this condition.

## 1 Introduction

Laparoscopic surgery provides feasible diagnostic and therapeutic abilities and is less invasive compared to conventional laparotomy. Currently, its feasibility and decreased invasiveness have been well established in most elective abdominal surgeries [1,2] and in some kinds of abdominal emergencies, such as appendicitis and cholecystitis [3,4]. Furthermore, it is gaining widespread acceptance in more major abdominal emergencies such as perforated peptic ulcers and small bowel obstruction [5,6]. We encountered two patients with small bowel perforation due to an ingested foreign body (FB)—a rare abdominal emergency—in whom laparoscopic surgery was successfully performed. In this case report study, we have emphasized the validity of laparoscopic surgery as an alternative in this rare abdominal emergency.

This work has been reported in accordance with the SCARE criteria [7].

## 2 Presentation of case

### 2.1 Case 1

**Fig. 1:**
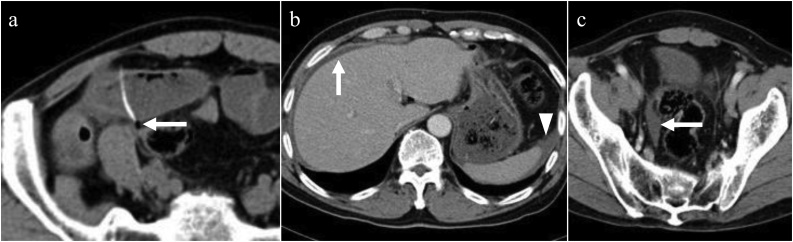
Findings from the computed tomography scan of the abdomen in Case 1. (a) A linear hyperdense foreign body (FB) inside the ileal lumen and free abdominal air adjacent to the FB (arrow). (b) Ascites around the liver (arrow) and the spleen (arrowhead). (c) Ascites in the pelvic cavity (arrow).

**Fig. 2:**
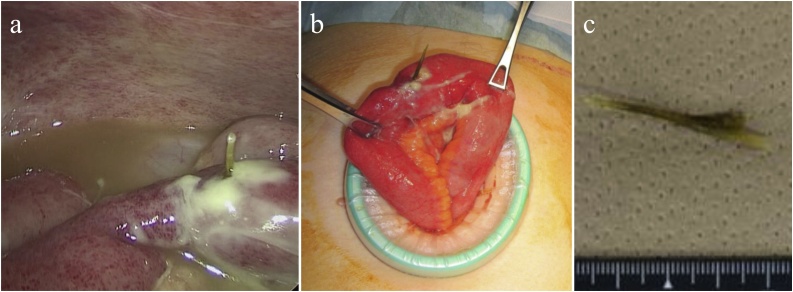
Intraoperative photograph and macroscopic findings in Case 1. (a) Intraoperative photograph showing turbid ascites and a foreign body (FB) piercing the ileal wall. (b) The perforation site was exteriorized via mini-laparotomy. (c) The FB was identified to be a fish bone.

A 75-year-old healthy man was brought in by an ambulance to the emergency department of our hospital with lower abdominal pain since 4 h with increasing severity. Abdominal examination revealed rebound tenderness and guarding around the lower abdomen. Computed tomography (CT) scan revealed a linear 35-mm hyperdense FB inside the ileal lumen, which had pierced the ileal wall at both ends (Fig. 1a). Additionally, free abdominal air adjacent to the FB and ascites around the liver, spleen, and in the pelvic cavity were observed (Fig. 1a, b, c). On questioning the patient, he reported of regularly consuming fish bones and having consumed fish a few days ago. A diagnosis of ileal perforation from a fish bone was presumed and emergent surgery was performed by board-certified surgeons. Mini-laparotomy was performed with a 50-mm vertical skin incision at the umbilicus, and an access device equipped with two 5-mm ports was inserted into the abdominal cavity via the incision. Thereafter, carbon dioxide pneumoperitoneum was induced, and a 5-mm rigid laparoscope and laparoscopic straight forceps were inserted. Laparoscopic observation revealed turbid ascites spreading extensively in the abdominal cavity (Fig. 2a). The small bowel was traced using the forceps and the FB perforation site was detected (Fig. 2a). Subsequently, the site was grasped and exteriorized via mini-laparotomy (Fig. 2b). Detailed observations using direct-viewing and palpation revealed two perforation sites at both the mesenteric and serosal sides of the ileum. Partial resection of the affected ileum was performed, followed by a hand-sewn end-to-end anastomosis. Following sufficient intra-abdominal lavage, drains were laparoscopically placed and the mini-laparotomy was closed cosmetically. An approximately 35-mm sharp fish bone was extracted from the resected ileum (Fig. 2c). The patient’s postoperative recovery was without complications, such as paralytic ileus, intra-abdominal abscess, and wound infection. He began oral intake and walking on the 2nd postoperative day (POD) and was discharged on the 10th POD.

### 2.2 Case 2

**Fig. 3:**
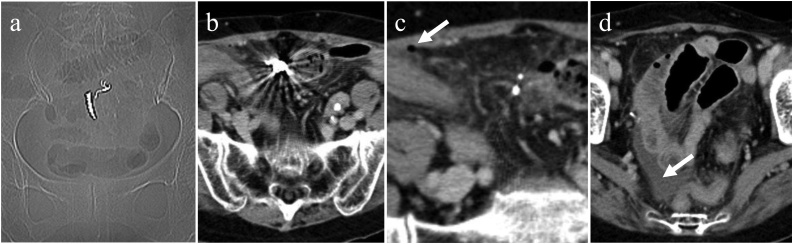
Findings from the radiograph and computed tomography (CT) scan of the abdomen in Case 2. (a) Radiograph showing a denture in the lower abdomen. (b) The denture was imaged as a hyperdense foreign body (FB) in the CT scan. (c) Abdominal free air (arrow) near the FB. (d) Ascites in the pelvic cavity (arrow).

**Fig. 4:**
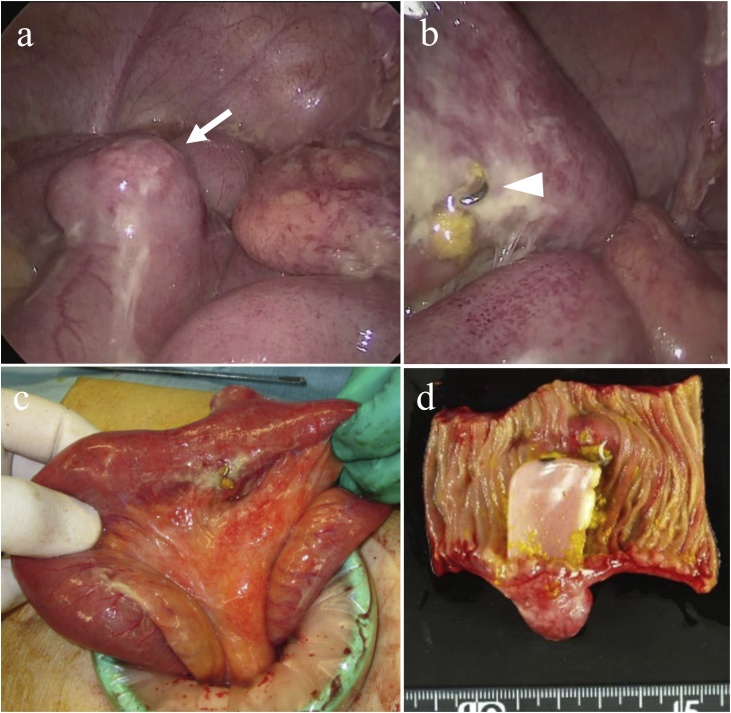
Intraoperative photograph and macroscopic findings in Case 2. (a, b) Intraoperative photograph showing the remarkably swollen part of the ileum (arrow) and perforation caused by a metallic component of the denture (arrowhead). (c) The perforation site was exteriorized via mini-laparotomy. (d) Dissection of the affected ileum revealed impaction of the denture in the ileal lumen.

A 71-year-old woman, with no relevant medical history, was brought in by an ambulance to our hospital with a 1-day history of increasing lower abdominal pain. On arrival, she reported of accidental ingestion of her denture three days ago. Physical examination revealed rebound tenderness and guarding around the lower abdomen. Radiography and CT scan revealed a hyperdense FB corresponding to the ingested denture in the lower abdomen (Fig. 3a, b). Additionally, CT scan revealed free abdominal air near the denture and ascites in the pelvic cavity (Fig. 3c, d). A diagnosis of small bowel perforation by the ingested denture was presumed and emergent surgery was performed by board-certified surgeons. As in Case 1, mini-laparotomy and pneumoperitoneum were established. Turbid ascites was observed in the pelvic cavity. Tracing of the small bowel revealed a remarkably swollen part of the ileum, which appeared to contain the denture (Fig. 4a) and perforation caused by the metallic component of the denture (Fig. 4b). Subsequently, the swollen ileum was exteriorized by mini-laparotomy (Fig. 4c). Partial resection of the affected ileum and reconstruction were performed, as in Case 1. Intra-abdominal lavage, drain placing, and wound closure were also similarly performed. Dissection of the affected ileum revealed impaction of the denture in the ileal lumen (Fig. 4d). The postoperative recovery of the patient was uneventful. She started walking on the 1 st POD and began oral intake on the 2nd POD. She was discharged on the 9th POD.

## 3 Discussion

The accidental ingestion of FBs, especially dietary FBs such as fish and chicken bones, is common and most of them pass through the gastrointestinal tract harmlessly within a week. However, gastrointestinal perforation is a rare complication, occurring in less than 1% of the cases of FB ingestion [8,9]. The clinical presentation can be greatly varied because it is affected by factors such as the site of perforation and the extent of spillage of the intestinal contents. Additionally, patients rarely have an awareness of having ingested FBs. Therefore, preoperative diagnosis of this disease can be challenging [10,11]. CT scan is supposed to be the most effective modality in the diagnosis of this condition [12,13] However, due to the following limitations, it may be overlooked: CT scan is not sensitive for radiolucent FBs, FBs may not be visualized on a conventional CT reconstruction with thick slices, and a lack of observer awareness can lead to an oversight in cases with poor images of FBs. These limitations result in the inadequate diagnostic ability of CT scans; hence, the probability of intra-operative diagnosis is high [10,12]. In the case of acute abdomen of an unknown etiology, maintaining a high index of suspicion and inquiring about accidental ingestion are very important.

Currently, laparoscopic surgery is gaining popularity in more abdominal surgical diseases because it has viable diagnostic and therapeutic abilities and is less invasive compared to conventional laparotomy. Its feasibility and advantages, such as less postoperative pain and faster recovery, have already been well established in the majority of elective abdominal surgeries [1,2], and in limited emergent settings including appendicitis and cholecystitis [3,4]. Furthermore, in more major abdominal emergencies, such as perforated peptic ulcers and small bowel obstruction, the superiority of laparoscopic surgery has been reported several times in literature [5,6]. Although confirming the effectiveness of laparoscopic surgery in small bowel perforation due to an ingested FB is difficult because of limited clinical experience, some case report studies described the feasibility and advantages of laparoscopy in this condition [11,[14], [15], [16], [17], [18]]. Furthermore, a few case series studies have favorably evaluated laparoscopy; albeit, these dealt with spontaneous small bowel perforation. Ding et al. described 15 patients with perforated Meckel’s diverticulum in whom laparoscopic management was performed and concluded that laparoscopy was a safe and effective surgical modality for both diagnosis and treatment and resulted in an excellent cosmetic result [19]. Sinha et al. described 20 patients with typhoid ileal perforation in whom laparoscopic intracorporeal bowel repair was performed and concluded that laparoscopic intervention was feasible and yielded favorable outcomes, especially in regard to limiting wound complications [20]. These studies suggest that laparoscopic management is also beneficial in the treatment of small bowel perforation due to an ingested FB; it contributed to the uneventful and fast recoveries of both patients in the present study.

Poor laparoscopic view, with such conditions as severe intestinal distension, adhesion, or intra-abdominal hemorrhage, can be a limitation of laparoscopy. These conditions may lead to inaccurate diagnosis, inappropriately prolonged operative time, and iatrogenic injuries. Conventional laparotomy should be considered for the safety of patients in cases with poor laparoscopic view.

If the surgeon’s skills and operative findings permit, pure laparoscopic surgery—in which all procedures, including repair of the perforation site, are completed under laparoscopic guidance—is a valuable alternative [11,16,18,20]. However, because the small bowel has good mobility, mini-laparotomy allows exteriorization of the perforation site and detailed observations using direct-viewing and palpation. When successive surgical procedures, such as partial resection are required, they require less time if performed under direct vision. Furthermore, these procedures do not require expensive laparoscopic instruments such as staplers. Hence, laparoscopy-assisted surgery accompanying mini-laparotomy for this condition is a reasonable choice [14].

## 4 Conclusion

Laparoscopic surgery has recently gained popularity in various kinds of abdominal emergencies; this technique can also be a valid alternative for small bowel perforation due to an ingested FB—a rare abdominal emergency. Furthermore, we advocate that laparoscopy-assisted surgery along with mini-laparotomy is the treatment of choice especially in this condition, as it is safe, fast, and cost-effective.

## Declaration of Competing Interest

All authors have no conflicts of interest.

## Sources of funding

This work was not supported by any funding agency.

## Ethical approval

This study was approved by the ethics committee of Iwate Prefectural Iwai Hospital.

## Consent

Written informed consent was obtained from the patients for the publication of this case report and accompanying images. A copy of the written consent is available for review by the Editor-in-Chief of this journal on request.

## Author contribution

SI is the main author of this article; SI, TU, and KK performed clinical treatment including surgery; YT, SH, TA, KS, and HK reviewed the manuscript; all authors have read and approved the final manuscript.

## Registration of research studies

Research registry (5064).

## Guarantor

The guarantor for this study is Soichi Ito.

## Provenance and peer review

Not commissioned, externally peer-reviewed
